# Vitreous inflammatory factors and serous retinal detachment in central retinal vein occlusion: a case control series

**DOI:** 10.1186/1476-9255-8-38

**Published:** 2011-12-12

**Authors:** Hidetaka Noma, Hideharu Funatsu, Tatsuya Mimura, Shuichiro Eguchi

**Affiliations:** 1 Department of Ophthalmology, Yachiyo Medical Center, Tokyo Women's Medical University, Chiba, Japan; 2 Department of Ophthalmology, University of Tokyo Graduate School of Medicine, Tokyo, Japan; 3 Department of Ophthalmology, Eguchi Eye Hospital, Hakodate, Japan

## Abstract

**Background:**

This study investigated whether the vitreous fluid levels of soluble vascular endothelial growth factor receptor-2 (sVEGFR-2), pigment epithelium-derived factor (PEDF), and soluble intercellular adhesion molecule 1 (sICAM-1) were associated with the occurrence of serous retinal detachment (SRD) in patients with central retinal vein occlusion (CRVO).

**Methods:**

We recruited 33 patients with CRVO and macular edema, as well as 18 controls with nonischemic ocular diseases. Eighteen of the 33 patients with CRVO showed SRD on optical coherence tomography of the macula (defined as subretinal accumulation of fluid with low reflectivity), while the other 15 patients only had cystoid macular edema (CME, defined as hyporeflective intraretinal cavities). Retinal ischemia was evaluated by measuring the area of capillary non-perfusion using fluorescein angiography and the public domain Scion Image program, while central macular thickness (CMT) was examined by optical coherence tomography. Vitreous fluid samples were obtained during pars plana vitrectomy and levels of the target molecules were measured by enzyme-linked immunosorbent assay.

**Results:**

Ischemia was significantly more common in the SRD group (17/18 patients) than in the CME group (5/15 patients) (*P *< 0.001). The vitreous fluid level of sICAM-1 increased significantly across the three groups from the control group (4.98 ± 1.73 ng/ml) to the CME group (15.4 ± 10.1 ng/ml) and the SRD group (27.1 ± 17.7 ng/ml) (*p_trend_*< 0.001). The vitreous fluid level of sVEGFR-2 also showed a significant increase across the three groups (1083 ± 541 pg/ml, 1181 ± 522 pg/ml, and 1535 ± 617 pg/ml, respectively, *p_trend _*= 0.019). On the other hand, the vitreous fluid level of PEDF showed a significant decrease across the three groups (56.4 ± 40.0 ng/ml, 24.3 ± 17.3 ng/ml, and 16.4 ± 12.6 ng/ml, respectively, *p_trend_*< 0.001).

**Conclusions:**

Higher levels of inflammatory factors (sICAM-1 and sVEGFR-2) and lower levels of anti-inflammatory PEDF were observed in macular edema patients with SRD, suggesting that inflammation plays a key role in determining the severity of CRVO.

## Background

Central retinal vein occlusion (CRVO) is a common retinal vascular disorder in patients with lifestyle-related diseases such as hypertension and atherosclerosis. The initiating event is thought to be thrombosis of the central retinal vein [1]. Occlusion of this major outflow channel for the retinal circulation markedly increases the intraluminal pressure within the retinal veins, resulting in hemorrhage and edema. Macular edema is the most common reason for impaired vision in patients with CRVO [2]. Optical coherence tomography (OCT) has demonstrated that macular edema secondary to CRVO is frequently associated with serous retinal detachment (SRD), cystoid macular edema (CME), and inner retinal thickening [3,4]. The reasons why SRD is associated with CRVO are unclear, but fluid leaking from damaged capillaries may migrate to the subretinal space and cause serous detachment. There is an increase of vascular endothelial growth factor (VEGF) secretion when acute vascular occlusion occurs in the retina [5]. We previously reported that VEGF and interleukin-6 (IL-6) are involved in the pathogenesis of SRD associated with CRVO [6], while Park et al. found that the aqueous humor level of VEGF was higher in patients with branch retinal vein occlusion (BRVO) and SRD than in those with BRVO and CME [7]. These finding suggest that inflammatory factors are associated with the occurrence of SRD in CRVO patients.

There is evidence that upregulation of various inflammatory factors, including vascular endothelial growth factor (VEGF), VEGF receptor-2 (VEGFR-2), intercellular adhesion molecule (ICAM)-1, and interleukin (IL)-6, and/or downregulation of anti-inflammatory factors like pigment epithelium-derived factor (PEDF), which is a potent inhibitor of angiogenesis [8], result in an increase of leukocyte-endothelial interactions that contribute to breakdown of the blood-retinal barrier (BRB) [9-11]. Blocking these inflammatory factors has been shown to prevent retinal leukostasis and an increase of retinal vascular permeability in rats [9]. In addition, occurrence of macular edema in patients with CRVO is associated with elevation of cytokines involved in regulation of the inflammatory response [11]. Although various inflammatory cytokines are reported to influence vascular permeability in the eye and to be related to macular edema in patients with CRVO, there is little evidence regarding the relationship of SRD to inflammatory molecules such as soluble VEGFR-2 (sVEGFR-2), soluble ICAM-1 (sICAM-1), and PEDF. sVEGFR-2 is produced as a result of alternative splicing of VEGFR-2 mRNA by retinal cells such as retinal glial Müller cells, and is a functional and soluble form of VEGFR-2 that lacks part of the intracellular domain [12]. ICAM-1 is normally produced by retinal pigment epithelium cells [13], and after adhesion molecules are shed by cells, its soluble form (sICAM-1) can be detected in serum and body fluids (including the vitreous fluid) [14]. sICAM-1 is formed by the five extracellular immunoglobulin domains of membrane-bound ICAM-1-after cleavage of these domains from the cell surface, possibly by a matrix metalloproteinase related to TNFα-converting enzyme or a human leukocyte elastase [15]. PEDF is produced by retinal cells such as retinal glial Müller cells and retinal pigment epithelial cells [8,10]. We performed the present study to investigate whether vitreous fluid levels of sVEGFR-2, sICAM-1, and PEDF were associated with SRD in patients who had CRVO.

## Methods

### Subjects

This study was performed in accordance with the Helsinki Declaration of 1975 (1983 revision). The institutional review boards of Tokyo Women's Medical University and Eguchi Hospital approved the protocol for collection and testing of vitreous fluid. Written informed consent was obtained from each subject following an explanation of the purpose and potential adverse effects of the procedure. Pars plana vitrectomy was performed at Tokyo Women's Medical University and Eguchi Hospital. Consecutive patients presenting with CRVO between October 2007 and March 2011 were screened according to the following inclusion and exclusion criteria. The inclusion criteria for this study were (1) CME (including SRD) secondary to CRVO in patients scheduled for pars plana vitrectomy, including patients who had received retinal photocoagulation, and (2) best-corrected visual acuity worse than 20/50 before surgery. Exclusion criteria were (1) previous ocular surgery or vitreous injection of anti-VEGF agents and triamcinolone acetonide, (2) diabetic retinopathy, (3) iris rubeosis, and (4) a history of ocular inflammation or vitreoretinal disease. Patients with iris rubeosis were excluded because it has been reported that they have high levels of cytokines [16], and the pathology of iris rubeosis may be different from that of macular edema. Patients with vitreous injection of anti-VEGF agents and triamcinolone acetonide were excluded because the molecules (including VEGF) may be affected by anti-VEGF agents and triamcinolone acetonide. Vitreous fluid samples were obtained from 33 CRVO patients and 18 patients with non-ischemic ocular disease (control group). These 18 patients without CRVO all had macular holes, but none of them had associated proliferative vitreoretinopathy. The CRVO patients comprised 16 men and 17 women aged 70.7 ± 8.2 years (mean ± SD), while the 18 patients in the control group included 10 men and 8 women aged 69.7 ± 9.5 years. The mean duration of CRVO was 4.9 ± 2.7 months (range: 2 - 12 months). Hypertension was diagnosed in patients being treated with antihypertensive agents or those who had a blood pressure > 140/90 mmHg [17]. As a result, 21 of the 31 patients (63.6%) had hypertension. Before surgery, panretinal photocoagulation was done to prevent neovascular glaucoma in 12 eyes (mean: 1,156 shots; range: 598 to 1,801 shots).

All patients underwent complete ophthalmic examinations including visual acuity, slit-lamp examination, funduscopy, FA, and optical coherence tomography (OCT) 3 (Stratus model 3000; Carl Zeiss, Dublin, CA). Best-corrected visual acuity was measured by Snellen visual acuity, which was converted into logarithm of the minimum angle of resolution (logMAR) for statistical comparison.

### Fundus Examination

Patients were evaluated by careful biomicroscopic examination using a fundus contact lens. The fundus findings were confirmed preoperatively by standardized fundus color photography and fluorescein angiography (FA), which was performed with a Topcon TRC-50EX fundus camera, an image-net system (Tokyo Optical Co. Ltd., Japan), and a preset lens with a slit-lamp.

The preoperative and operative fundus findings were recorded for each subject. A masked grader independently assessed ischemic occlusion from fundus photographs. Areas of retinal photocoagulation were excluded when calculating the size of the non-perfused region. When the non-perfused area divided by the disc area yielded a value of 10 or more, this was defined as indicating the presence of retinal ischemia [18-20].

We divided patients into two groups according to the presence of SRD confirmed by OCT examinations [7]. Serous retinal detachment was defined as typical subretinal fluid accumulation in neurosensory retinal detachment with low or absent reflectivity anterior to a clearly distinguishable outer band irrespective of the coexistence of CME. We defined CME if there were hyporeflective intraretinal cavities on OCT images. The central macular thickness (CMT) was measured by OCT at scan lengths of 6.0 mm using a fast macular thickness map. Central macular thickness was defined as the distance between the inner retinal surface and the retinal pigment epithelium including SRD. Thickness of neurosensory retina was defined as the distance between the inner and the outer neurosensory retinal surfaces [21]; these were measured using the calipers on the OCT machine.

### Sample Collection

Samples of undiluted vitreous fluid (500 - 1,000 μl) were collected at the start of vitrectomy by aspiration into a 1 ml syringe attached to the vitreous cutter before commencing the intravitreal infusion of balanced salt solution. The vitreous samples were immediately transferred into sterile tubes and were rapidly frozen at -80°C.

### Measurement of sICAM-1, sVEGFR-2, and PEDF Levels

Levels of sICAM-1 were measured in vitreous fluid samples (from the same eye) as well as in plasma with enzyme-linked immunosorbent assay using kits for human sICAM-1 (Bender Med Systems, Burlingame, CA, USA) [22]. PEDF was measured in vitreous and plasma samples from the same patients with a human PEDF Sandwich ELISA kit (Chemicon International, Temecula, CA) [23]. Vitreous fluid levels of sVEGFR-2 were also determined by ELISA according to the manufacturer's instructions (Mitsubishi Chemical Medience Corporation, Tokyo, Japan) [24]. A volume of 100 μL of assay diluent RD 1W (R&D Systems) and 100 μL of either the standard control or a 5-fold diluted vitreous fluid sample were added to each well of the ELISA plate, which was incubated for 2 hours on a shaker at room temperature and washed. Then a horseradish peroxidase-labeled polyclonal antibody for sVEGFR-2 was added to each well, followed by incubation for 2 hours with shaking at room temperature and washing of the plate. Next, the substrate solution (200 μL), color reagent A (hydrogen peroxide; R&D Systems), and color reagent B (chromogen; R&D Systems) were mixed together in equal volumes and added to each well, after which the plate was incubated for 30 minutes at room temperature in darkness. Finally, 50 μL of stop solution (2N sulfuric acid; R&D Systems) was added, and the absorbance was determined at 450 nm (with correction at 540 or 570 nm) by using a microplate reader. The levels of these molecules in the vitreous fluid were within the detection ranges of the assays, with the minimum detectable concentration being 3.3 ng/ml for sICAM-1 (intra-assay coefficient of variation (CV): 5.4%, inter-assay CV: 7.8%), 1.95 ng/ml for PEDF (intra-assay CV: 5.4%; inter-assay CV: 7.9%) and 78.1 pg/ml for sVEGFR-2 (intra-assay CV: 5.5%, inter-assay CV: 6.9%).

### Statistical Analysis

All analyses were performed with SAS System 9.1 software (SAS Institute Inc., Cary, North Carolina, USA). Data are presented as frequencies or as the mean ± SD. Student's *t*-test was employed to compare normally distributed unpaired continuous variables between the two groups and the one-way-ANOVA was used among three groups. Normality of the distribution of data was assessed with the Kolmogorov-Smirnov test. The chi-square test or Fisher's exact test was used to compare nominal variables. To assess the trends of sVEGFR-2, sICAM-1, and PEDF levels among the groups, a linear regression model was used. A two-tailed P value of less than 0.05 indicated statistical significance.

## Results

A profile of the subjects is given in Table 1. The 33 CRVO patients were classified into 15 with CME (CME group) and 18 with SRD (SRD group). The mean age and the female/male ratio were similar among the three groups (*P *= 0.900 and *P *= 0.874, respectively), but there was a significant difference in the prevalence of hypertension (*P *= 0.017). The duration of CRVO was similar in the CME and SRD groups (*P *= 0.704). The incidence of ischemia was significantly higher in the SRD group than in the CME group (*P *< 0.001), but performance of panretinal photocoagulation did not show a significant difference between these two groups (*P *= 0.064).

**Table 1:** Clinical Features of Patients With CRVO

Findings	Control (N = 18)	CME (N = 15)	SRD (N = 18)	P-value
Age (years)	69.7 ± 9.5^‡^	71.1 ± 8.7^‡^	70.3 ± 8.1^‡^	0.900

Gender(female/male)	8/10	8/7	9/9	0.874

Hypertension	4	10	11	0.017

Duration of CRVO (months)	N/A	5.2 ± 2.9^‡^	4.8 ± 2.6^‡^	0.704

Ischemia	N/A	5	17	< 0.001

Panretinal photocoagulation	N/A	8	4	0.064

CRVO = central retinal vein occlusion; CME = cystoid macular edema; SRD = serous retinal detachment

^‡ ^Mean ± standard deviation (SD).CRVO = central retinal vein occlusion. N/A = NotApplicable.

**Figure 1:**
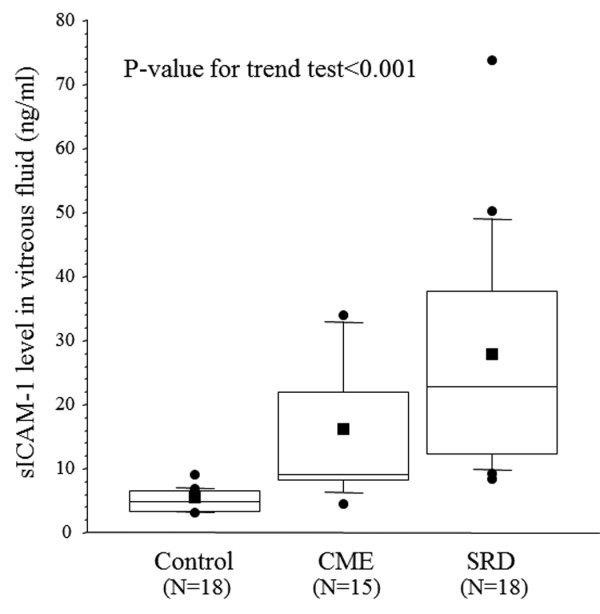
**Vitreous fluid levels of soluble intercellular adhesion molecule-1 (sICAM-1) in the CRVO patients and controls**. Horizontal bars in the box plots display 10%, 25%, 50% (median), 75%, and 90%, while the square plot indicates the mean value for sICAM-1.

**Figure 2:**
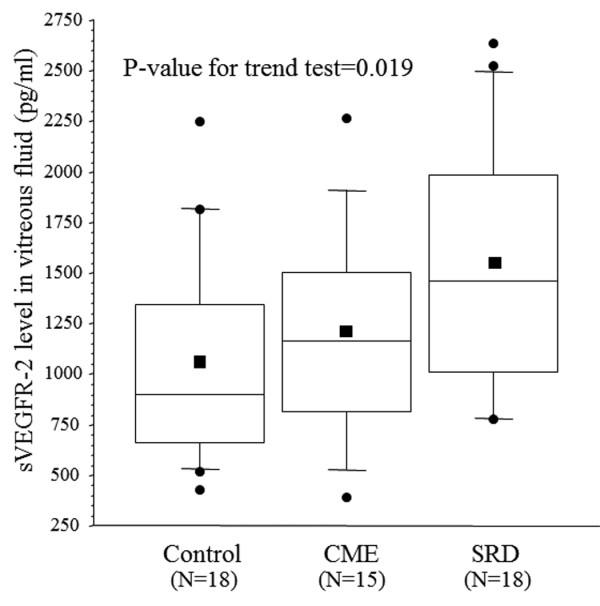
**Vitreous fluid levels of soluble vascular endothelial growth factor receptor-2 (sVEGFR-2) in the CRVO patients and controls**. Horizontal bars in the box plots display 10%, 25%, 50% (median), 75%, and 90%, while the square plot indicates the mean value for sVEGFR-2.

**Figure 3:**
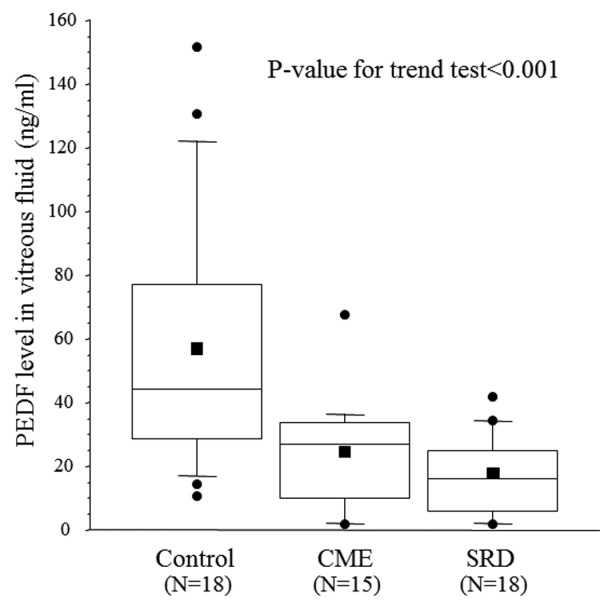
**Vitreous fluid levels of pigment epithelium-derived factor (PEDF) in the CRVO patients and controls**. Horizontal bars in the box plots display 10%, 25%, 50% (median), 75%, and 90%, while the square plot indicates the mean value for PEDF.

There was a significant increase in the vitreous fluid level of sICAM-1 across the three groups from the control group (4.98 ± 1.73 ng/ml) to the CME group (15.4 ± 10.1 ng/ml) and the SRD group (27.1 ± 17.7 ng/ml) (*p_trend_*< 0.001; Figure 1). There was also a significant increase in the vitreous fluid level of sVEGFR-2 across the three groups (1083 ± 541 pg/ml, 1181 ± 522 pg/ml, and 1535 ± 617 pg/ml, respectively, *p_trend _*= 0.019) (Figure 2). Conversely, the PEDF level showed a significant decrease across the three groups (56.4 ± 40.0 ng/ml, 24.3 ± 17.3 ng/ml, and 16.4 ± 12.6 ng/ml, respectively, *p_trend_*< 0.001) (Figure 3).

No significant differences were noted with regard to the vitreous levels of sICAM-1, sVEGFR-2, or PEDF between the 12 patients who received preoperative retinal photocoagulation and the 21 patients who did not (*P *= 0.396, *P *= 0.125, and *P *= 0.176, respectively).

## Discussion

The present study demonstrated a significant increase in the vitreous fluid level of sICAM-1 across the three groups from the control group to the CME group and the SRD group. It has been reported that CME is the most common form of macular edema secondary to CRVO, and SRD is often found to be associated with CME in CRVO patients who undergo OCT [3,4]. Vascular leakage from congested veins or capillaries could be a major source of subretinal fluid and SRD secondary to CRVO may arise from transudation of intraretinal extracellular fluid into the subretinal space [3,4]. The foveal architecture probably also has an influence, especially the Müller cell cone [4]. In the present study, retinal ischemia was significantly more common in the SRD group than the CME group, suggesting that retinal ischemia may influence the occurrence of SRD. We previously reported that an increase of intraocular sICAM-1 is associated with increased vascular permeability and with more severe retinal ischemia in CRVO patients [22]. These findings and the present results suggest that increased vascular permeability due to upregulation of sICAM-1 may contribute to SRD in patients with CRVO, as has already been demonstrated for VEGF [6]. In addition, increased ICAM-1 expression by endothelial cells would increase leukocyte migration and result in the accumulation of sICAM-1 in serum and body fluids such as the vitreous fluid [14]. The results of the present study suggest that leukocyte migration is a feature of SRD. Therefore, an alternative explanation is that systemic athrosclerosis or vasculitis causes CRVO, after which sICAM-1 enters the vitreous fluid due to increased vascular permeability. Thus, sICAM-1 entering the vitreous fluid could contribute to the complications of CRVO in patients with athrosclerosis or vasculitis, but further investigation would be required to confirm this.

The present study also identified a significant increase in the vitreous fluid level of sVEGFR-2 across the three groups from the control group to the CME group and the SRD group. It has been reported that activation of VEGFR-2 contributes to disruption of tight junctions by decreasing occludin expression [25]. In addition, Ojima et al. [26] reported that VEGF-induced phosphorylation of VEGFR-2 and activation of downstream signaling are related to breakdown of the BRB. Furthermore, suppression of VEGFR-2 signaling inhibits VEGF-mediated loss of tight junction proteins in retinal endothelial cells and blocks the VEGF-mediated increase of retinal vascular leakage [27]. Moreover, VEGFR-2/Src kinase inhibitors have been shown to prevent a VEGF-induced increase of vascular permeability and reduce retinal edema in an experimental model of retinal ischemia [28]. Such inflammatory effects of sVEGFR-2 may contribute to an increase of retinal vascular permeability. We previously reported that the product of vitreous sVEGFR-2 and VEGF levels (sVEGFR-2 × VEGF) was significantly correlated with the severity of macular edema in CRVO patients, although the vitreous fluid level of sVEGFR-2 alone was not [24]. This suggests that an increase of sVEGFR-2 along with up-regulation of VEGF is associated with more severe macular edema. Accordingly, retinal vascular permeability may increase because of the upregulation of both sVEGFR-2 and VEGF, contributing to the development of SRD in patients with CRVO.

Finally, we found that the PEDF level showed a significant decrease across the three groups from the control group to the CME group and the SRD group. In contrast, Park et al. [7] reported no significant difference in the aqueous humor level of PEDF between SRD and CME patients. However, measurement of these molecules in vitreous fluid is more accurate for investigating changes of retinal physiology than measurement in aqueous humor, which could account for difference between our findings and those of Park. PEDF is a negative acute-phase protein, so an increase of PEDF would probably inhibit retinal inflammation and prevent breakdown of the BRB. Intravitreal injection of PEDF was reported to significantly reduce retinal vascular permeability in an animal model, along with an increase in the retinal level of occludin, which is a major component of endothelial tight junctions [10]. In the same model, the retinal levels of inflammatory factors (including VEGF and ICAM-1) were significantly lower in PEDF-treated eyes than in control eyes [10]. In cultured retinal endothelial cells, PEDF ameliorates the VEGF-induced increase of permeability, consistent with *in vivo *findings [29]. In addition, retinal Müller cells show a significant increase of VEGF production after silencing of PEDF [10]. These results and the present findings suggest that PEDF acts as an endogenous inhibitor of inflammation which blocks the expression and activity of various inflammatory molecules, thus contributing to a reduction of vascular permeability. Accordingly, an increase of vascular permeability secondary to downregulation of PEDF may contribute to the development of SRD in patients with CRVO. In addition, the above-mentioned findings taken together with the results of this study suggest that PEDF may normally inhibit retinal inflammation (control group) and prevent breakdown of the BRB, while PEDF expression seems to be reduced in CRVO patients.

In the present study, we found high levels of inflammatory factors like sICAM-1 and a low level of anti-inflammatory PEDF in CRVO patients with SRD, suggesting that inflammation is active in these patients. Therefore, anti-VEGF therapy that only reduces the ocular level of free VEGF may be inadequate for CRVO patients with SRD. Addition of triamcinolone acetonide could be more effective because it modulates vascular permeability by downregulating inflammatory factors (ICAM-1 [30] and VEGFR-2 [31]) and by upregulating anti-inflammatory factors such as PEDF [32]. Thus, assessing the pathologic features of each patient (such as the presence or absence of SRD) may allow us to select the best treatment strategy for macular edema associated with CRVO, but a randomized prospective clinical trial of each therapy would be required to confirm this.

The present study had the following limitations. The first is that some of our subjects received laser treatment, which could have influenced the vitreous fluid levels of sVEGFR-2 and VEGF. Although we found no significant difference of vitreous sVEGFR-2 and VEGF levels between the 12 patients with preoperative retinal photocoagulation and the 21 patients who did not receive it, further investigation will be required to confirm the influence of retinal photocoagulation on vitreous fluid levels of sVEGFR-2 and VEGF. Second, we performed vitrectomy for macular edema because it has been reported that vitrectomy can improve macular edema and visual acuity in CRVO patients [33,34]. However, current standard care for CRVO includes anti-VEGF agents since the CRUISE trial showed improvement of visual acuity with ranibizumab therapy [35]. This suggests that vitrectomy should probably be considered following the failure of anti-VEGF therapy, but such a treatment strategy for CRVO needs to be investigated in the future. Third, there is no evidence how changes of various molecules (including VEGFR-2) in the vitreous fluid are reflected in the retinal tissues because the function of sVEGFR-2 in the vitreous remains unknown. Further studies of VEGFR-2 in this disease would be needed to determine whether its kinetics in the vitreous fluid actually reflect those in the retinal tissues of CRVO patients.

## Conclusions

In conclusion, we found that the vitreous fluid levels of sICAM-1 and sVEGFR-2 were higher in CRVO patients who had SRD compared with those who had CME, while the vitreous PEDF level was lower in patients with SRD. The higher levels of inflammatory factors (sICAM-1 and sVEGFR-2) and lower levels of anti-inflammatory PEDF observed in macular edema patients with SRD suggest that inflammation may have an important influence on the severity of CRVO.

## Competing interests

The authors declare that they have no competing interests.

## Authors' contributions

HN, and HF were involved in the design and conduct of the study. Collection and management of the data were done by H.N., and S.E., while analysis and interpretation of the data were performed by H.N., T.M., and S.E. Preparation of the first draft of the manuscript was done by H.N., and review and approval of the manuscript was performed by H.F., and T.M. All authors have read and approved the final manuscript.
